# Taking a Stand for Office-Based Workers' Mental Health: The Return of the Microbreak

**DOI:** 10.3389/fpubh.2020.00215

**Published:** 2020-06-11

**Authors:** Casey Peter Mainsbridge, Dean Cooley, Sarah Dawkins, Kristy de Salas, Jiajin Tong, Matthew Wade Schmidt, Scott J. Pedersen

**Affiliations:** ^1^School of Education, Launceston, TAS, Australia; ^2^School of Education, Ballarat, VIC, Australia; ^3^Tasmanian School of Business and Economics, Hobart, TAS, Australia; ^4^School of Technology, Environments, and Design, Hobart, TAS, Australia; ^5^School of Psychological and Cognitive Sciences, Beijing Key Laboratory of Behavior and Mental Health, Beijing, China; ^6^School of Health Sciences, Hobart, TAS, Australia

**Keywords:** occupational health, mental health, microbreaks, stress, fatigue, vigor, prolonged sitting

## Abstract

There is evidence that movement-based microbreaks can improve the cardiovascular health of desk-based employees, but their effect on mood states is yet to be investigated. As daily work tasks can potentially result in the loss of physical and psychological resources, the objective of this study was to measure the effect of movement microbreaks during formal work time on mood states. In a randomized-controlled pilot study with repeated measures (baseline, post-test, washout) of self-reported job stress and mood states (fatigue and vigor), police officers (*N* = 43) were exposed to movement microbreaks during work hours. A multivariate significant difference between groups was noted after the intervention period. Further analysis revealed that the experimental group reported a latent reduction in job-related stress after the 3-months washout period. Although the study was conducted with a small sample, our preliminary findings suggest that interrupting sedentary work with movement microbreaks may have beneficial effects on employee mental health. The implications of movement microbreaks for mitigating work-related stress of first responders, including police, is discussed, along with directives for future research.

## Introduction

Technology in the workplace has altered the pace, intensity, efficiency, and duration of office-based work. The advent of internet and devices such as smart phones (1) has led to changes in working practices such as 24-h access, teleworking, hot desking, and flexitime (2, 3). One unintended outcome of these changes is reduced opportunity for physical movement at work, which has resulted in prolonged periods of sitting at work, particularly in desk-based roles. The flow on effect is a concomitant rise in cumulative trauma disorders (CTD) (4). CTD are a range of health complaints such as stress, pain, mood swings, and fatigue that underpin more serious diseases. Research indicates that CTD have negative long-term effects on health status (5–8). Moreover, the changes in workflow due to technology advances has seen the loss of the microbreak (9). The microbreak is a short, informal break which can occur spontaneously throughout the workday (10, 11). Microbreaks are associated with reducing the incidences of CTD (4) because the break is taken in response to a perceived loss of a resource, such as an inability to maintain attention or change in mood state (12). Microbreaks therefore provide an opportunity to improve perceptions of stress and mood state in desk-based workers. The aim of this study was to reintroduce the microbreak back into desk-based work to determine its effect on workers' affective states.

Employees can experience job-related stress due to a range of stressors such as excessive or undue work demands, management of their own work responsibilities, tasks of their own, and pressure to meet objectives (13). Subsequently, employees can suffer from personal difficulty, strain, anxiety, and worry in attempts to countering such stressors. In a study (14) composed of government, private, and non-government organizations, employees reported high work demands, low-control over work situations, effort-reward imbalance related to working conditions, and management style were the primary causes of work-related stress (15). Stress emanating from work can contribute to psychological distress, physical, and mental illness (16–18). It is generally acknowledged by employers and employees that stressful work environments at times can be unavoidable. Consequently, organizations implement a range of therapeutic interventions to aid employees to recover from work demands and creating healthy work-life balance. A strategy from such programs is the use of work breaks as a key to combatting work stress (15).

Apart from CTD, work stress can also be manifested through physical and emotional responses, which negatively impact upon psychological factors such as mood. Mood can be measured using five negative states; “tension-anxiety,” “depression-dejection,” “anger-hostility,” “fatigue-inertia,” “confusion-bewilderment,” and one positive state, “vigor-activity” (19). Each state has a bi-directional relationship or hedonic flexibility principle, indicating employees can change their mood by engaging in a range of activities or by changing their environments (20). For example, sunshine and higher temperatures made travel mood more positive and relaxed for vehicle and public transport users but led to negative mood for cycling and walking commuters in three different Swedish cities (21). Conversely, an inability to maintain the ideal mood state has been associated with stress (22, 23) and adverse health symptomology (24, 25).

Following the hedonic flexibility principle, mood states impact on employees' choices of activities. Specifically, employees use their mood as a resource (26, 27). When employees feel good, they can endure tasks which they find tedious, such as completing repetitive work tasks. When employees find themselves feeling mentally fatigued, they can swap and engage in different activities which can alter their mood. Low energy-based activities that incorporate movement and physical action are associated with a range of mood alterations. For example, interrupting occupational sitting by standing and walking within the workplace to talk to a colleague is associated with a corresponding elevation in positive moods (12, 28–30). Similarly, a brief session of yoga in the workplace resulted in a reduction in the negative mood state of fatigue, and a concurrent increase in employee's positive mood state of vigor (31). Increasing energy expenditure activities also reflect the hedonic flexibility principle with one daily 15-min session of aerobic exercise resulting in attenuated mood states for anger and hostility, as well as depressive symptomology (32). A more comprehensive Bosster Break intervention (including aerobic exercises, strengthening exercises, and flexibility exercises) resulted in reports of reduced stress, increases in enjoyment and health awareness, facilitated behavior change, and enhanced workplace social interaction (33). Comparably, changes of the work environment also are associated with mood alterations. For instance, university students who physically moved to view a flowery meadow roof scene, compared to a concrete scene, reported improved attention, attention control, and vigor (11). These findings provide evidence to suggest that a change to the environment by including some form of movement has a positive effect on employees' mood states, specifically reversing negative mood states to be more positive.

Despite these positive associations, there are shortcomings when applied to workplace settings. Primarily interventions and approaches to date have incorporated movement breaks into the workday by means of a single break and for a continuous period of time (such as 15 min). In some cases, this might not be possible in all workplaces, such as in call centers, with emergency contact response employees, reception and first point of contact employees, information technology employees, air traffic control employees, and occupations that are performed primarily through a computer. Moreover, the use of traditional forms of physical activity exclude non-leisure time exercisers. There is some evidence that non-leisure time exercisers are willing to engage in non-exercise physical activity (NEPA). In a series of studies (34–39) of Tasmanian government employees classified as non-leisure time exercisers, elected to incorporate into hourly prompted microbreaks throughout a normal workday. In these studies, employees engaged in movement microbreaks were operationally defined as low-intensity, short-duration, NEPA. NEPA were comprised of movements that allowed incorporation into the normal daily work routine. For example, standing up from a seated position to take a telephone call; or taking the stairs, rather than taking the elevator, to attend a meeting. Although participation was voluntary, once in the study, employees had hourly prompted software installed on their work computers to take a micro-movement break. Employees were able to self-select the type of movement, the duration, and repetitions. Results revealed high adherence and compliance rates over 13 weeks (37, 39). Moreover, the samples self-reported increases in daily energy expenditure (36), and perceptions of quality of life (38); with associated reductions in blood pressure (34, 35). These results suggest that NEPA might also be associated with changes in mood state, especially in a population that rejects traditional forms of leisure time exercise.

The focus of this study was to investigate if microbreaks comprised of regular, low-dose NEPA, would alter desk-based employees' mood states and perceptions of job-related stress. In particular, we were interested in the mood states of vigor and fatigue as there is evidence that participation in physical activity is associated with increases and decreases, respectively (34–39). We were also interested to explore if the movement microbreaks would ameliorate perceptions of stress associated with work tasks. Based on a lack of literature related to the impact of low-dose, movement microbreaks on measures of mental health status, in this pilot study we tested the null hypothesis that regular movement microbreaks would not significantly change desk-based employees' mood states and perceptions of organizational stress.

## Method

### Research Design

We conducted a pilot quasi-experimental, field-based, repeated measures (pre-intervention, post-intervention, washout) research design with random assignment with replacement to an experimental or control group. The experimental group received the movement microbreak software (39) on their work computers for 13 weeks during the intervention period. Both groups were followed for an additional 13 weeks after the intervention was removed from the experimental group (washout period).

### Participants

The participants of this study were identified within the Tasmanian Department of Police and Emergency Management (TDPEM). Policing environments are diverse, with stressors emanating from a combination of danger, ambiguity, human misery, and death, yet also involve stressors such as ineffective workplace organizational structures and operational processes such as shift work, excessive overtime and poor communication (40, 41). Unsurprisingly, police employees disproportionally experience poorer mental and physical health than the general population (41, 42). With a mission to deliver policing services to build a safe, secure, and resilient Tasmania, the TDPEM understood that many of their employees spend a considerable amount of their time sitting isolated in front of desktop computers and sought an opportunity to engage them in improving their workplace mental health.

Participants for this field-based, randomized-control pilot study were selected from a state-wide population of TDPEM employees. The structure of this organization included 70 Police stations spread across the state, each varying in size and infrastructure. All police employees were contacted by the TDPEM occupational health and safety officer through email. The email contained health information about prolonged bouts of sitting and an invitation to use the intervention designed by the research team to prompt seated employees to stand up every hour to engage in a self-selected movement microbreak. The researchers received a 25 per cent positive return (*N* = 91). To adequately power the study, we selected a stratified sample with equal representation from the employment regions of the organization based on the percentage of desk-based employees specific to that region. We deemed that a medium effect size would be meaningful for each dependent variable. *A priori* calculations for adequate participant numbers were set with power at 0.80, and α at 0.05, for a planned medium effect (*d* = 0.25), which indicated a total sample size of 76 was deemed sufficient.

From the initial number (*N* = 91) pool, the research team applied selection criteria; (1) full-time employee with primarily desk-based job responsibilities being available to complete the study requirements; (2) used a personal computer with internet access to perform work; (3) classified as a non-exerciser (<30 min of exercise per week for a period of 3 months), were prepared to engage in behavior change (43); (4) were deemed medically healthy via a PAR-Questionnaire (44) to perform the self-selected, movement microbreaks suggested by the software; and (5) available for a 6-months study including baseline, post-test (after 13 weeks) and washout (after 26 weeks) data collection points. This screening excluded 48 employees because of their inability to meet all the selection criteria (ethics #H0010875).

**Figure 1:**
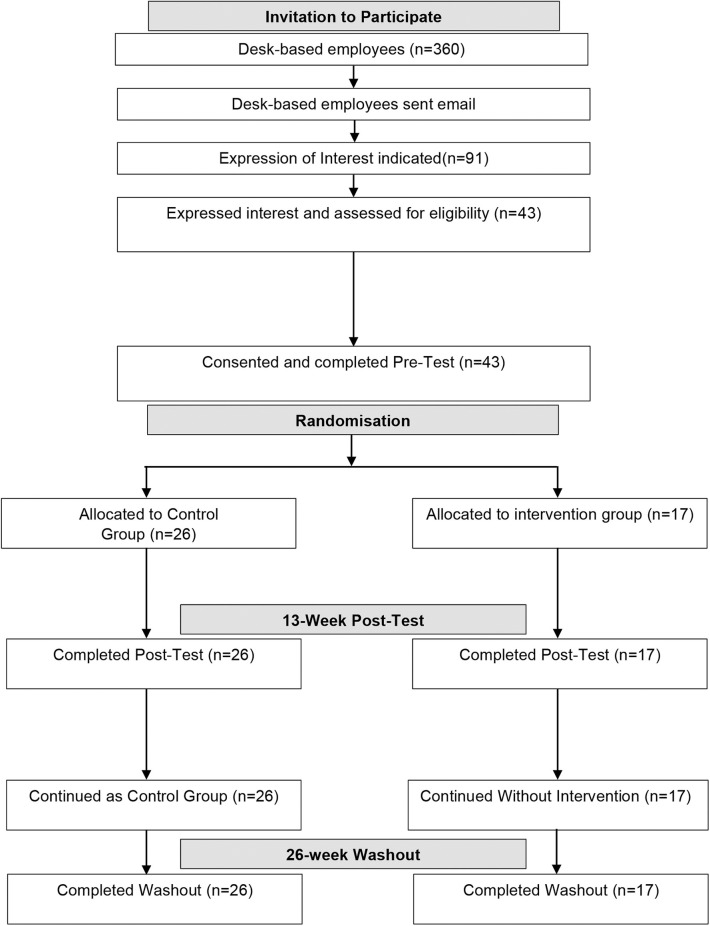
Consort flow diagram of invitation to participate, group, allocation and follow-up.

After the application of selection criteria, 43 employees (32 females and 11 males; *mean age* = 42.52 ± 10.89) were included in the study and subjected to randomization software to select the experimental groups. All excluded participants were informed of the reasons for non-selection into the study and were provided with the intervention at the conclusion of the study. Types of occupations included in this investigation were receptionist, administrative support, call center, forensic analysis, community liaison, media liaison, transcription, and tech support. Demographic data were all electronically self-reported during work hours (Table 1), as were the data collected on the scales for the dependent variables described below. A flow diagram for invitation to participate, group, allocation, and follow up is presented in Figure 1.

**Table 1:** Participant demographic data.

**Gender (*N* = 43)**	**Age (years)**	**Weight (kg)**	**Height (cm)**	**BMI**
Female (*n* = 32)	41.69 (12.07)	72.69 (13.82)	164.09 (6.68)	26.98 (4.71)
Male (*n* = 11)	44.27 (6.84)	98.27 (17.73)	178.45 (3.64)	30.80 (5.01)

*Values are means (standard deviations)*.

### Measures

To assess the impact of the workplace intervention on participants' mood states and self-reported job stress, two measures were employed electronically: a Police Stress Questionnaire (45) and the Profile of Mood States (POMS) inventory (19).

### Police Stress Questionnaire

Stress was measured using the Police Stress Questionnaire (45). This 40-item self-report questionnaire contains two subscales: operational stressors relating to job content, such as field work; and organizational stressors relating to job context, such as clerical work. The Police Stress Questionnaire is scored as a cumulative sum for each subscale, with higher scores reflecting greater perceived stress during work. In this pilot study only the organizational stress subscale (PSQ-Org) was employed, as the population of interest were administrative and not involved in police operational duties (i.e., arrests, foot and traffic patrols, special operations teams). To measure perceived organizational stress derived from environmental sources, participants responded to 20 items regarding their experience of stress at work over the past 3 months on a 7-point Likert scale anchored from (1) “no stress at all” to (7) “a lot of stress.” Individual items were summed for the PSQ-Org and reported as mean and standard deviation. Example items included “too much computer work” and “If you are sick or injured your co-workers seem to look down on you.” The original authors reported acceptable coefficients for validity (*r*^2^ values ranging from 7 to 22%) and internal reliability (α = 0.92) for the PSQ-Org (45). In the present study, the internal consistencies for perceived stress were 0.93, 0.92, and 0.94 for the three data collection time points, respectively.

### Profile of Mood States (POMS) Inventory

Two of the subscales of the POMS inventory are related to changes in mood or feelings of energy: the energy-specific vigor-activity (POMS-Vigor: *n* = 8 items) subscale for positive mood, and the fatigue-inertia (POMS-Fatigue: *n* = 7 items) subscale to measure negative mood (24, 46, 47). Participants completed these two subscales presented individually on 5-point Likert-type scales (0 = “Not at all” to 5 = “Extremely,”). Items were summed separately for each subscale and reported as means and standard deviations. Higher scores indicate higher levels of the mood states (e.g., energy-specific vigor-activity and fatigue-inertia) that participants experienced during work hours over the past week. We selected the stem, “In the last 7 days” rather than a shorter time period because we were interested in assessing if the intervention had a sustainable effect on mood states rather than transient effects. Suitable measures of internal consistency reliability (α > 0.80) and concurrent validity (α > 0.74) have been previously reported for these POMS subscales (46, 47). In the present study, the internal consistencies for both subscales were acceptable (POMS-Fatigue = 0.96 for both baseline and post-test, and 0.97 for the washout test; and POMS-Vigor = 0.94 for baseline and 0.95 for both post-test and washout test).

## Procedures

### Pre-intervention Phase

The research team conducted an orientation session with all participants. The purpose of this session was to discuss using the movement microbreak intervention during work hours. Baseline data collection and experimental group allocation for our field-based trial were also accomplished during this session. During the orientation session participants were informed of possible health effects associated with prolonged sitting at work and provided strategies for interrupting sitting during the workday. The last portion of the session was dedicated to trialing the movement microbreak software. Some participants asked questions about being away from their computer, or what to do during meetings and video conferences. These participants were reassured that movement microbreak prompts were just prompts and engaging in the healthy behavior suggestions during work was an individual discretion. There were no restrictions placed on frequency or intensity during the prompted hourly microbreaks. Once all questions were answered, baseline data were collected through a digital web-based survey tool. Average time to complete the online survey was 12 ± 2.34 min.

After baseline data were collected participants were randomly assigned with replacement to either an experimental group (*n* = 17; 82% Female; *Mean*_*Age*_= 40.18 ± 12.94 years) who had the intervention installed on their work computers, with next day implementation; or a control group (*n* = 26; 70% Female; *Mean*_*Age*_ = 43.77 ± 9.44 years) who continued to work as normal. All participants were asked not to make any changes to other aspects of their lifestyle during the 26-weeks experimental period such as starting any other new exercise programs, well-being strategies, or fad diets. Those participants randomly assigned to the control group were informed that they would receive the intervention once the six-month study period was over.

### Intervention Phase

The intervention involved a prompting sequence to encourage participants to rethink their decision to remain seated after 60 min of computer work. The prompt was a small window that appeared in the lower right hand of the computer screen indicating that 60 min of continual computer work had elapsed, and the microbreak screen was going to initiate. At this point, participants could choose to engage the microbreak selection sequence immediately; or postpone the sequence once for 15 min. At the end of this 15-min interval, the microbreak selection sequence screens covers the employee's entire computer screen preventing the continuance of computer work. This screen displays until participants complete a movement microbreak of their choice (e.g., chair squats) and record their progress. At this point, the hostage screen disengages, and participants can access their working screen(s). The decision for an hourly prompt time was based on national guidelines for office employees (48).

The microbreak selection screen contained 65 different NEPA choices (e.g., stair climb, stork stand, walking), with digital video coaching. All participants were informed during the orientation session that the decision on type of activity, duration, and intensity was an individual choice. However, the prompt was passive in delivery during the first 3 months (in that participants did not need to engage in responding to the system), thereby forcing participants to engage with the intervention on an hourly basis. During the 13-weeks intervention period, each movement microbreak was date/time stamped by the software once employees recorded their progress (activity specific—either in terms of the number of repetitions or duration in seconds). This daily progress could be optionally viewed by participants as bar graphs, measured in caloric expenditure and non-sedentary time, at the end of each movement microbreak sequence. On average, aggregate daily use of the software self-reported by the experimental group was 7.21 ± 2.56 times per workday.

### Post Intervention Phase

After the 13-week intervention period baseline measures were repeated and reported as post-test data. At this time, the movement microbreak software was removed from all computers. After a second 13-weeks period (washout) the baseline measures were repeated once more.

### Data Analysis

To examine if there were significant differences in predicting the three dependent variables (vigor, fatigue, & organizational stress) between experimental and control groups at post-test and after the washout period, we conducted a one-way multivariate analysis of variance (MANOVA) after controlling for baseline scores, age, and gender. Significant multivariate findings were followed up with univariate ANOVA procedures including simple main effects and independent sample *t*-tests for *post hoc* analysis. *A priori* alpha levels were set at 0.05 for all inferential tests of significance. Due to the pilot nature of this investigation and the underpowered sample size, to control against type 2 error effect sizes (η*2* and Cohen's *d* statistic) were reported for the appropriate statistical analyses. All data were analyzed using PASW version 18.0 (49).

## Results

The multivariate, mixed design analysis suggested a significant between-group difference at post-test (Wilk's? = 0.79, multivariate *F*_(3,34)_ = 3.09, *p* = 0.04, η^2^ = 0.21), but not at washout (Wilk's?=0.85, multivariate *F*_(3,34)_ = 2.08, *p* = 0.12, η^2^ = 0.16). To examine the function of the movement microbreak intervention, we further compared group differences at each time point on each outcome variable. We proceeded with three separate univariate ANOVA using a 2 (group: experimental, control) X 3 (time: baseline, post-test, washout) mixed design ANOVA separately for the three dependent variables (PSQ-Org, POMS-Fatigue, and POMS-Vigor).

In predicting perceived stress (PSQ-Org), the ANOVA results showed a significant interaction between group and time after controlling for age and gender, *F*_(2,78)_ = 4.21, *p* = 0.02, η^2^ = 0.10. Follow-up analysis revealed no significant differences between the groups for baseline and the post-test (*d* = 0.36 ± 0.36 & 0.21 ± 0.35, *t* = 1.02 & 0.60, *p* > 0.10), but a significant difference between groups during the washout test (*d* = 0.85 ± 0.38, *t* = 2.23, *p* = 0.03), with a medium effect size (*Cohen's d* = 0.77, 95% *CI* = 0.14~1.40). Thus, we rejected our null hypothesis that police officers allocated to desk-based duties who interrupted their occupational sitting would not experience a reduction in self-reported stress stemming from their organizational environment, compared to their colleagues who maintained their normal desk-based occupational patterns.

Univariate analyses for mood profile changes revealed no significant interactions between group and time for fatigue (POMS-Fatigue), *F*_(2,78)_ = 1.39, *p* = 0.25, η^2^ = 0.04, or for vigor (POMS-Vigor), *F*_(2,78)_ = 1.92, *p* = 0.15, η^2^ = 0.05, after controlling for baseline, age, and gender. Group mean differences for the three dependent variables are indicated in Table 2.

**Table 2:** Descriptive statistics for the control and experimental groups across time.

**Variable**	**Group**	**Baseline**	**Post-test**	**Washout**
	Experimental	2.23 (1.11)	2.40 (1.08)	2.12 (1.06)*
Perceived stress				
	Control	2.66 (1.13)	2.59 (1.10)	3.03 (1.23)
	Experimental	3.17 (0.88)	3.62 (0.61)	3.38 (0.70)
Vigor				
	Control	2.81 (0.86)	2.89 (0.93)	2.89 (0.91)
	Experimental	1.93 (0.70)	1.67 (0.47)	1.85 (0.66)
Fatigue				
	Control	2.32 (1.06)	2.35 (1.08)	2.40 (1.17)

Values are Likert scale means (standard deviations). Group mean difference (*p < 0.05).

## Discussion

Our pilot study findings indicated that desk-based employees engaged in desk-based work who were exposed to sustained and regular prompts to complete low dose NEPA (i.e., microbreaks) demonstrated a significant interaction between job-related stress and the mood states of vigor and fatigue. The directional movement of these three combined dependent variables in the experimental group over time indicates that perceptions of stress and mood states can be positively affected by using targeted movement microbreaks designed to instigate interruptions to sitting posture, and then have employees engage in some form of physical activity. This multivariate analysis suggested that both stress and mood variables have a possible influence on the other. Despite being pilot in nature and being low in power, this novel finding requires further exploration. With no previous literature to refer to within this experimental design it is difficult to speculate on the meaning of this multivariate finding. Nonetheless, previous research in the workplace has acknowledged the inter-relationship between stress, depression, and anxiety, and the various effects these variables can have on health broadly (15). Thus, we followed this analysis with separate univariate analysis.

Our novel finding has implications for the health of desk-based workers who suffer from stress and negative mood states (50). Workplace stress can adversely impact components of mood states such as anxiety, fatigue, and depression, thus decreasing stress in the workplace may improve mood over the long-term possibly leading to employees feeling positive (50). Cautiously, it would appear that our microbreak strategy significantly decreased job-related stress compared to their counterparts who maintained their regular occupational sitting habits. Notwithstanding, changes in perceptions of stress are not easily realized through short-term, non-therapeutic interventions (51–53). Thus, our intervention strategy offers the first evidence that organizational stress can possibly be attenuated by having desk-based workers take a break from their tasks to engage in some self-determined movement activity. Second, despite the evidence that employees' probability of signing up and adhering to workplace programs is related to prior exercise habits, time costs, taste for fitness, confidence, and positive attitude toward fitness (54, 55) our intervention realized a positive outcome for adherence and compliance in a non-exercising population. We argue that this result was achieved by having movement break activities that were self-determined, office-appropriate, did not require specialized equipment or a change of clothing, and were short in duration. This finding has potential implications for the design of future workplace health and wellbeing studies, especially those which target individuals who are most likely to experience the largest effect as a result of initiating a movement-based program (56, 57).

There are some notes of caution for the stress-related results reported in this study. The use of a self-report to determine perceptions of stress rather than a biological measure is open to subjective error (58). For example, some individual Likert scale items had standard deviations >2. Moreover, the stem for each item on the inventory directed participants to think about stress in the previous 3 months (45). There are memory and positivity-bias issues with using self-recall data (59). Although different in regard to time (immediate vs. recall) a biological measure taken at the time of the microbreak would have provided an instantaneous measure of how participating in NEPA microbreaks not only aided in offsetting the physical effects of prolonged sitting but also had a concomitant effect of stress. We would suggest that future designs use a measure of salivary cortisol to determine a more precise interpretation of effect. Such evidence would reduce the subjectivity of our findings and allow for the generalization of our data toward the development of targeted workplace policies and practices.

Despite the multivariate interaction of the three dependent variables, when measured independently both mood state variables did not exhibit significant differences between groups across time points. A possible explanation for the non-interaction is the high reactivity of mood to environmental and personal experiences and the possible latency associated with change in mood (20, 59). For example, mood state is influenced by many different factors (i.e., time of day, presence of others, hunger) and hence a single measure of mood profile on any given day or time might have been confounded by an immediate reaction present in the environment not measured in this study. Moreover, during the washout period, the change to environment (e.g., prompted microbreaks) initiated by the software had ceased for participants, so it could be somewhat expected that once the prompt had ceased, any benefit to mood would also stop. The use of a mood diary (60, 61) in combination with the current study design could potentially counter this limitation for future research.

Similarly, there is debate about the various methods used to measure mood (62, 63). It is acknowledged that from both a physiological and cognitive standpoint that accurately and consistently capturing valid and reliable data can be biased toward emotionally salient information that reflects one's emotional state at that present moment (61). Whilst arguments that self-report instruments are acceptable for measuring certain psychological states such as mood and stress (64), objective measures (e.g., biomarkers such as cortisol) might provide a more immediate temporal link to participation in microbreaks and changes in mood states.

Overall, our pilot study provides preliminary evidence for the consideration of ‘old wine in a new bottle' policy, the return of microbreaks to workflow (65). Moreover, designing microbreaks to include regular, low-dose, movement-based activities to help improve or sustain employees' health. Such a policy would be advantageous in that it would be inclusive of more technology into the workplace, increased changes to work, while providing a mechanism to maintain good mental health. In this study, the advent of regular movement-based microbreaks during work hours resulted in a self-reported latent decrease in job-related stress. We suggest to further evaluate this finding that future field-based work include a washout period and be conducted for longer duration (e.g., >6 months). Finally, a comment on the use of persuasive technology driven behavior change. Future field-based research should be mindful that persuasive technologies can suffer from a lack of adoption (66, 67), particularly during work hours. One reason for this can be attributed to a lack of theoretical understanding of human behavior change that these technologies are being developed to impact upon. If technologists engage with theory, for example the Behavior Change Wheel (BCW) (68) to inform the content and process within their technology, this would improve its suitability for the target behaviors. The BCW describes a methodology by which intervention designers can systematically examine the behaviors the intervention aims to target. BCW then has recommendations for target audiences to enhance engagement, efficacy, opportunity and motivation (68). Our method, while not informed by the BCW, reflects this approach. If technical developers incorporate some of the elements of our method into their technology designed to change target behaviors, they are likely to increase the robustness of the technology and its capacity to achieve its goal of behavior change.

## Data Availability Statement

The raw data supporting the conclusions of this article will be made available by the authors, without undue reservation.

## Ethics Statement

The studies involving human participants were reviewed and approved by Tasmania Social Sciences Human Research Ethics Committee, reference number H0018075. The patients/participants provided their written informed consent to participate in this study.

## Author Contributions

SP, DC, and CM conceived the presented idea, carried out the experiment, collected the data for this study, and took the lead in writing the manuscript. SP, DC, CM, SD, KS, and JT developed the theory and performed the calculations and analysis of the data. SP, DC, CM, and JT verified the statistical and analytical methods. SD, KS, MS, JT, SP, DC, and CM contributed to the interpretation of the results. SP, DC, and CM wrote the manuscript with support from SD, KS, and MS. SP, DC, CM, and SD contributed to the final version of the manuscript. All authors discussed the results and commented on the manuscript design and presentation, provided critical feedback and helped shape the research, analysis, and manuscript.

## Conflict of Interest

The authors declare that the research was conducted in the absence of any commercial or financial relationships that could be construed as a potential conflict of interest.
